# Expanding the Utilization of Robotic Procedures in Urologic Surgery

**DOI:** 10.5041/RMMJ.10320

**Published:** 2017-10-16

**Authors:** Tareq Aro, Michael Mullerad, Gilad E. Amiel

**Affiliations:** 1 Urology Department, Rambam Health Care Campus, Haifa, Israel; 2 Rappaport Faculty of Medicine, Technion–Israel Institute of Technology, Haifa, Israel

**Keywords:** Laparoscopy, robotic surgery, urology

## Abstract

Although the word “robot” was coined in 1921, only close to 70 years later were robotic devices developed to assist during surgery. Urology has always been at the forefront of endoscopic, minimally invasive, and robotic developments in medicine. Robotic prostatectomy signaled the emerging role of robotic surgery in urology, but since then it has been applied to every urologic laparoscopic procedure.

## INTRODUCTION

Karel Čapek first coined the word “robot” in his play *R.U.R. (Rossum’s Universal Robots)*, in 1921.^1^ The term was derived from the Czech word “robota,” meaning “forced work.” Čapek’s play presented a world where humans were assisted by robots to fulfill everyday tasks; however, the robots eventually turned on their masters and sought world domination.^2^

“A computer-controlled manipulator with artificial sensing that can be reprogrammed to move and position tools to carry out a range of surgical tasks” was the definition given to a surgical robot.^3^ Many authors did not like this definition and suggested “computer-assisted surgery” as an alternative.^4^

Invention has always been driven by necessity, and so was the development of medical robotics. EndoAssist, probably the first surgical robotic device, was introduced in 1990 and was a free-standing laparoscopic camera manipulator, controlled by infrared signals from a headset worn by the surgeon.^2^ The first robot-assisted human hip replacement was performed in California using a Robodoc in 1992.^5^

Automated Endoscopic System for Optimal Positioning (AESOP) was manufactured by Computer Motion and used voice (or pedal) control.^6^ AESOP™ 1000 in 1993 was the first commercially used robotic assistant, used to hold the endoscopic camera in laparoscopic surgery, and was shown to be steadier and more effective than human assistance.^7^

The ZEUS™ Robotic Surgical System used in 1998 consisted of three robotic arms attached to the side of the operating table and controlled with a hand-held joystick. This was the first system allowing a surgeon to control laparoscopic instruments. The ZEUS™ Robotic Surgical System was used for the first transatlantic surgery in 2001, when a surgeon in New York performed cholecystectomy on a patient in France.^8^

The da Vinci Surgical System developed by Intuitive Surgical made a giant leap in the use of robotics in surgery by bringing the most advanced “master–slave” system developed. The basic principle involves control of three or four robotic arms by a surgeon sitting at a console. The system has three components: a surgeon console, a patient-side cart, and an image-processing stack.^2^

In the year 2000 there were reports of about 1,500 robotic surgeries worldwide, and this number increased to more than 20,000 by the year 2004.^5^ While the initial reports of robotic surgery were in the field of cardiac surgery, the vast majority of these procedures are performed in urology nowadays. Most notable is radical prostatectomy, where the procedure was performed robot-assisted in about 10% of cases in the United States in 2006^2^ and almost entirely performed robotically in the United States nowadays. In the last decade, urologic surgeons have constantly expanded the use of the robot for increasingly complex procedures. Our goal was to survey those cases and describe the expansion of robotic surgery in urology.

## METHODS

An online PubMed search for key words involving robotic surgery, urology, or the name of specific procedures was performed. Also, a targeted search for robotic surgery and technologies publications in the last two American Urology Association meetings was undertaken.

## HISTORIC MILESTONES IN ROBOTIC UROLOGIC SURGERY

Urology has always been at the forefront of endoscopic, minimally invasive, and robotic developments in medicine. Robotic prostatectomy signaled the emerging role of robot surgery in urology, but since then it has been applied to every urologic laparoscopic procedure. The only factor that hindered progress of robotics at its beginning was the initial high costs of the device itself.

The advantage of robotic over conventional laparoscopic procedures is obviously more pronounced in procedures requiring complex reconstructive techniques, and so the simpler procedures, even though they can be performed robotically, are likely to be performed using other techniques, including free hand laparoscopy.

### Adrenalectomy

The adrenals are situated in a deep anatomic position, which is ideal for a laparoscopic approach, and so the robotic approach presents obvious advantages. Horgan et al.^9^ were the first to perform robotic laparoscopic adrenalectomy in humans in 2001. By 2003 there were several reports of the advantages of using the robotic approach.^10^,^11^ However, most adrenalectomies being performed today are still through the laparoscopic approach.

### Renal Surgery

#### Simple and Radical Nephrectomy

Laparoscopy remains the most widely used method for both simple and radical nephrectomy to date. The first laparoscopic nephrectomy was described by Ratner et al. in 1995.^12^ The ease of the operation, and the lack of complex maneuvers, makes the use of the robot unnecessary. In 2005 Klingler et al.^13^ described the first robotic nephrectomy.

#### Partial Nephrectomy

As our understanding of renal cell carcinoma grew larger, the main surgical treatment of localized lesions today is partial nephrectomy compared to the previous gold standard—radical nephrectomy.

There were clear benefits for the laparoscopic approach to the deeply positioned kidney that is surrounded by several vital structures from every direction.

The main problem with the laparoscopic approach for partial nephrectomy was the need for difficult maneuvers and suturing which was responsible for a challenging operation with a steep learning curve.

The first robotic partial nephrectomy was published in 2005.^14^ The introduction of the robotic system made this surgery less challenging to perform. By 2013, 64.1% of partial nephrectomies in the USA were performed robotically.^15^

#### Vena Cava Thrombectomy

After gaining enough experience in robotic renal surgery, surgeons felt comfortable performing more complicated procedures, including the evacuation of tumors extending from the kidney into the vena cava. The first robotic nephrectomy with an inferior vena cava (IVC) tumor extension was in 2008 and the first series published in 2011.^16^ Nonetheless, the vast majority of level II and above vena cava thrombectomy cases are still performed in an open fashion.

#### Nephroureterectomy

The standard treatment of upper-tract transitional cell carcinoma is nephroureterectomy with bladder cuff excision. Several reports demonstrated similar results between the open and laparoscopic technique. The learning curve for performing the operation laparoscopically was too difficult to make it a widespread technique, even though it was shown to be feasible.^17^,^18^ Robotic nephroureterectomy was first reported in 2006 by Rose et al.^19^ Nonetheless, the majority of cases are probably still performed via a laparoscopic approach.

#### Live-donor Nephrectomy

The first series of robotic live-donor nephrectomies was reported in 2001 by the University of Illinois at Chicago.^9^,^20^ However, just like radical nephrectomy, most current reported series are performed via the laparoscopic approach.

#### Kidney Transplantation

The first attempted robotic kidney transplantation was performed in France in 2001 but was not performed fully using the robot.^20^ Since then there have been many reports and publications trying to perfect the technique, and the first fully robotic transabdominal transplantation was reported in 2010.^21^

The disadvantages of the robot included the need for methods to cool the kidney and pelvis to prevent longer warm ischemia time and reports of longer times to creatinine clearance improvement, which is thought to be attributed to the positive pressure during pneumoperitoneum.

Still, there is value in the robotic approach especially in morbidly obese patients who were otherwise denied kidney transplant due to increased risk of surgical site infection.^22^

#### Pyeloplasty

Laparoscopic dismembered pyeloplasty was first performed in 1993, and further reports showed similar success rates to open pyeloplasty. The laparoscopic approach remains a challenging procedure even in larger series.^23^ The increased dexterity and precision make robotic pyeloplasty an attractive alternative option. The robotic approach is used both in adults and in the pediatric population.^24^–^26^

### Ureteral Surgery

All operations involving the ureter (whether distal or partial ureterectomy; reimplantation of the ureter; or primary ureter anastomosis) require advanced techniques that are extremely difficult to perform laparoscopically. The robotic system allows the surgeon to expand its ability to perform dissections and sutures similar to open surgery without the necessity for a large incision. Continuous sutures present an easier technique than interrupted in the laparoscopic and robotic settings.^27^–^29^ Musch et al. published in 2013 a series of a single-institution experiences with distal ureteral reconstructive surgery with good results.^30^

### Bladder Surgery

#### Cystectomy

The first robotic radical cystectomy was reported in 2003.^31^ The operation included performing a complete intra-abdominal formation of an orthotopic ileal neobladder.

By now many studies have debated the benefits of the robotic approach compared to open cystectomy. These demonstrated equivalent results to the open approach in terms of pathological outcomes and lymph node dissection. The robotic approach causes less blood loss with longer operative times.^32^–^34^

A randomized controlled trial published in 2015 showed similar 30-day and 90-day complication rates for the two approaches, with 90-day complication rates of 62% in the robotic group and 66% in the open approach.^32^

A review of 19 studies including 1,779 patients in 2015 demonstrated better outcomes with the robotic approach, demonstrating a greater lymph node yield, fewer perioperative complications, less blood loss and need of transfusions, and shorter hospital stays.^35^

Another review article comparing the two operations demonstrated the same results of better perioperative outcomes in the robotic group, with non-inferiority results in terms of oncological outcomes.^36^

By 2010 the number of robotic radical cystectomies in the USA had risen to 12.8%,^37^ and by 2015 it was published that most academic centers in the USA have adopted the robotic approach for the operation.^38^

We are currently anticipating the results of the multi-institutional RAZOR (randomized open versus robotic cystectomy) trial that started in 2014 and which may become a landmark in the future of radical cystectomy.^39^ We reported the first series of robotic cystectomy in Israel in 2015^40^ and a series of post-radiation salvage cystectomies in 2017.^41^

#### Partial Cystectomy

Partial cystectomy is a less common operation than the gold standard radical cystectomy for bladder cancer, but there are a few indications for performing partial cystectomy including for non-malignant causes. By the year 2010 the first reports of performing the operation robotically emerged.^42^ However, although feasible, it is not considered standard of care, and therefore no reports of large series have been published.

#### Bladder Augmentation

Bladder augmentation is performed mainly in patients with neurogenic bladder. The first complete laparoscopic ileal cystoplasty was reported in 2002 in an adult.^43^ By 2004 the first appendicovesicostomy (Mitrofanoff procedure) in a child was reported,^44^ and in 2008 the first robotic operation was published.^45^

### Urinary Diversion

In 1950, Bricker described the first ileal conduit.^46^ The Wallace method of uretero-ileal anastomosis was described in 1966.^47^ Many methods were described over the years for continent versus incontinent urinary diversions, and ileal conduit remains the most commonly used urinary diversion technique.^48^

To date, most of urinary diversions are still performed extracorporeally; still, there is a rising number of surgeons performing both types of diversions intracorporeally with advances in technique still being published.

### Prostate Surgery

#### Simple Prostatectomy

Mariano et al. in 2002 described the first laparoscopic simple prostatectomy for benign prostate enlargement and later on reported their 6-year experience.^49^,^50^ Due to its technical difficulty, the operation did not gain a lot of popularity.

In 2008, Sotelo et al.^51^ described the first robotic simple prostatectomy which became more popular than the laparoscopic approach.

#### Radical Prostatectomy

The hallmark of robotic surgery is still robotic radical prostatectomy. In 1991, Clayman and colleagues performed the first laparoscopic radical prostatectomy, which was published in their series of nine patients in 1997.^52^ In 2001, the Henry Ford Hospital described the first robotic radical prostatectomy.^53^ Since then the robotic approach has become the procedure of choice in the US and in many centers worldwide.

In July, 2016 the first randomized controlled trial comparing the open radical prostatectomy to robotic radical prostatectomy was published in *The Lancet*, showing superiority of the robotic approach in intraoperative complications, blood loss, hospital stay, and postoperative complications.^54^

#### Nerve Grafting

Despite advances in the technique of robotic radical prostatectomy, and despite the practice of nerve sparing, adequate cancer control may require sacrificing the neurovascular bundle during surgery. A number of studies have demonstrated the feasibility of immediate nerve reconstruction after prostatectomy. However, clinical outcomes have been mixed.^55^ One well-designed randomized controlled trial demonstrated no benefit of unilateral nerve grafting after prostatectomy.^56^

Even though results are still debatable and the need for the procedure remains questionable, in 2003 the first reports of robotic sural nerve grafting technique was published.^57^ Since then, however, sural nerve grafting has been abandoned completely.

### Retroperitoneal Lymph Node Dissection

Despite retroperitoneal lymph node dissection (RPLND) being one of the most challenging operations in urology to perform laparoscopically, some high-volume centers with dedicated surgeons perform RPLND, mainly primary rather than post chemo.^58^ In 2011 the first publication of robotic RPLND appeared.^59^ Recently, robotic surgeons started publishing their initial series with excellent results.^60^

### Microsurgery

In recent years there has been an expanding use of the robotic system for microsurgical procedures.

#### Vasectomy Reversal

The first evolution in infertility treatment came in 2004 with the first da Vinci microsurgical vasectomy reversal.^61^,^62^ Parekattil et al. reported similar outcomes comparing the robotic to the pure microsurgical technique.^63^ However, those results were in the beginning of the robotic era and without a skilled microsurgery assistant.^62^ Some recent publications show the benefit of the robotic approach in difficult anatomical situations, as in Trost et al. describing a bilateral intracorporeal vasovasostomy in a case of iatrogenic bilateral obstruction following hernia repair.^64^

#### Varicocelectomy

The first experience with subinguinal robotic-assisted varicocelectomy came in 2008,^65^ and Mechlin and McCullough published in 2013 good outcomes of the procedure along with an observation that this method provided a better controlled environment for training of residents and fellows.^66^

#### Testicular Sperm Extraction

Microsurgical testicular sperm extraction (TESE) has the highest sperm retrieval rate among the various retrieval methods for non-obstructive azoospermia.^67^ The procedure utilizes simultaneous imaging of the tissue by an embryologist which is further enhanced by the combination of the robotic system and new sperm imaging detection techniques.^62^

#### Targeted Denervation of the Spermatic Cord

The procedure is aimed at treating patients with chronic groin or scrotal pain. A 2014 publication demonstrated complete pain resolution in 70.5% of patients and significant improvement in 84.8% of patients.^68^

In a recent conference (RAMSES, Maastricht, March 2017) there were several video sessions on advancing urologic robotic microsurgical techniques, including microsurgical denervation of the spermatic cord, varicocelectomy, and vasectomy reversal procedures (see: ramsesrobotics.com).

## RECENT DESCRIPTION OF NOVEL TECHNIQUES

After almost 20 years of advancement in urologic robotic surgery, we continue to try and bring new technologies, perfect the different techniques, and discover various new indications for the use of the robotic laparoscopic systems in urologic practice.

In the last two American Urology Association (AUA) conferences held in San Diego in May, 2016 and in Boston in May, 2017, there were 282 and 365 publications on robotic surgery, respectively (647 in total), 397 of them (61%) related to oncologic surgeries. Table 1 stratifies all AUA 2016 and 2017 publications. It is also worth mentioning that nine presentations were on RPLND, six presentations on robotic sacrocolpopexy, and two presentations on robotic vaginoplasty.

**Table 1:** Stratification of all AUA 2016 and 2017 Publications.

Topic	Publications
Prostate	191
Kidney+Inferior Vena Cava	153
Bladder	65
Ureters	37
Bowel segments	24
Pediatrics	22
Genitourinary reconstruction	13
Adrenal	12
Urethra	8
Other	122

Out of 647 presentations relating to robotic surgery, 108 (17%) discussed new or improved techniques. Of those, 85 were video presentations, 18 were poster presentations, and only five were accepted for a podium presentation. Table 2 shows the stratification of the presentations dealing with innovative techniques.

**Table 2:** Stratification of AUA 2016 and 2017 Surgical Innovation Presentations.

Topic	Surgical Innovation Presentations
Prostate	41
Kidney+Inferior Vena Cava	29
Bladder	13
Ureters	8
Bowel	7
Others	4
Adrenal	3
Pediatrics	3

## CONCLUSION

Urology has been in the forefront of implementing and advancing robotic surgery in medicine. Since their development, robotic systems have been integrated into almost every aspect of urologic surgery. Even nearly 20 years after the appearance of the first robotic system, new technologies and indications are being published in increasing numbers annually. The last two AUA conferences included almost 650 publications involving robot-assisted procedures.

There are numerous reports about medical device companies that are at advanced stages of developing new robotic systems for treating kidney stones and as an alternative to existing devices. We hope that competition will make robotic technology more affordable, and as robot systems become more prevalent they will assume a leading role in the future of surgery in general and in urology in particular.

## Abbreviations

AUA: American Urology Association
IVC: inferior vena cava
RPLND: retroperitoneal lymph node dissection
TESE: testicular sperm extraction
